# Early and Very Early Post-Transcatheter Aortic Valve Implantation Infective Endocarditis: Imaging Challenges

**DOI:** 10.31083/RCM36771

**Published:** 2025-06-30

**Authors:** Nikoleta Hadjigeorgiou, Despo Melanarkiti, Theodora Eleni Plakomyti, Vasileios Demou, Vasileios Giannakopoulos, Chariton Sapouridis, Angeliki Mouzarou

**Affiliations:** ^1^Department of Cardiology, General Hospital Paphos, State Health Services Organization, 8026 Paphos, Cyprus

**Keywords:** very early post-TAVI infective endocarditis, early post-TAVI infective endocarditis, post-TAVI infective endocarditis

## Abstract

Transcatheter aortic valve implantation (TAVI) is a minimally invasive procedure to treat severe aortic stenosis in select patients. Patients who have undergone TAVI are at high risk of infective endocarditis (IE), especially during the first year post-operation. Early diagnosis of IE is essential to initiate targeted antibiotic therapy and/or surgical intervention. However, the early detection of IE following TAVI poses significant diagnostic challenges. Current imaging techniques, including echocardiography, nuclear imaging, and magnetic resonance imaging, have varying degrees of sensitivity and specificity, each with inherent limitations. Nuclear imaging modalities, such as positron emission tomography/computed tomography using ^18^F-fluorodeoxyglucose (^18^F-FDG PET/CT) and white blood cell single photon emission computed tomography/computed tomography (WBC SPECT/CT), have shown promise in early IE detection, particularly due to the ability of these methods to identify metabolic and anatomical abnormalities. However, false-positive results related to post-operative inflammation complicate data interpretation, and limited data exist for using these methods in very early IE detection post-TAVI. Intracardiac echocardiography (ICE) offers enhanced visualization of prosthetic valve leaflets, but the invasive nature of ICE restricts its widespread use. Whole-body imaging, such as ^18^F-FDG PET/CT, facilitates the identification of distant lesions and systemic complications, aiding diagnosis and treatment decisions. Diagnosing IE after TAVI is especially challenging within the first 60 days post-procedure, a critical period when imaging findings may be inconclusive due to false negatives or limited availability of advanced modalities. This review underscores the diagnostic complexity of very early and early (0–60 days) IE post-TAVI, emphasizing the need for a multimodal imaging approach to overcome the limitations of individual modalities. Nonetheless, early antimicrobial therapy should be considered even without definitive imaging findings, highlighting the importance of clinical vigilance in managing this challenging condition.

## 1. Introduction

Transcatheter aortic valve implantation (TAVI) has emerged as a vital, minimally invasive intervention for severe symptomatic aortic stenosis, particularly in older patients (aged >75) deemed high-risk for surgical aortic valve replacement (SAVR) [1]. The popularity of TAVI has surged recently, as this technique has expanded to include younger patient populations, prompting an expectation of even greater increases in TAVI procedures [2, 3, 4]. This trend is bolstered by ongoing randomized clinical trials, such as TAVR UNLOAD (NCT02661451), PROGRESS (NCT04889872), and the Evolut™ EXPAND TAVR II Pivotal Trial (NCT05149755), which aim to demonstrate significant survival benefits [5]. Although the TAVR UNLOAD trial did not show a significant survival advantage compared to conservative management in patients with moderate aortic stenosis and heart failure with reduced ejection fraction (HFrEF), it did highlight the potential for quality-of-life improvements, especially in symptomatic patients. As clinical understanding continues to evolve, there is a growing belief that even symptomatic moderate aortic stenosis may necessitate intervention [6].

Infective endocarditis (IE) refers to the inflammation of the endocardium, which is the inner lining of the heart. The intact, healthy endocardium is typically resistant to bacterial seeding [7]. However, the development of infectious endocarditis necessitates initial endocardial injury, followed by bacteremia [8]. Among the serious complications associated with TAVI, prosthetic valve endocarditis (PVE) poses a considerable risk, with studies revealing a one-year incidence of IE ranging from 0.2% (in the PARTNER 3 trial) to 3.1% (in a Danish cohort of 509 patients) [9, 10]. Both patient-related and procedural-related risk factors significantly contribute to the development of IE following TAVI [11].

The timing of IE post-TAVI, concerning the procedure, can be categorized as very early (within 30 days), early (within 30–60 days), intermediate (between 60 and 365 days), or late (after 365 days) [12]. Nonetheless, accurate diagnosis is crucial, as it dictates the initiation of targeted antibiotic therapy and potential surgical intervention [13]. However, diagnosing IE in TAVI patients can be particularly challenging due to the elusive nature of vegetation and the need for advanced imaging techniques [14]. The European Society of Cardiology (ESC) has modified diagnostic criteria incorporating additional imaging modalities to aid this endeavor [15, 16]. Meanwhile, imaging techniques such as transthoracic (TTE) and transesophageal echocardiography (TEE/TOE) remain the cornerstone for initial evaluation. However, additional modalities, including computed tomography (CT), cardiac computed tomography angiography (CTA), magnetic resonance imaging (MRI), and nuclear imaging such as positron emission tomography/computed tomography using ^18^F-fluorodeoxyglucose (^18^F-FDG PET/CT) and single photon emission tomography/computed tomography with white blood cell (WBC SPECT/CT) labeling, are becoming increasingly utilized. Furthermore, intracardiac echocardiography (ICE) is emerging as a valuable tool in this context. Despite modifications, diagnosing very early and early IE after TAVI remains challenging.

Given the elevated in-hospital and 30-day mortality rates associated with IE, largely attributable to advanced age and multiple comorbidities, early suspicion and diagnosis are paramount [17]. Antimicrobial therapy for IE post-TAVI is similar to that of PVE. Therefore, in cases where PVE is highly suspected, it is vital to initiate appropriate antibiotic treatment without delay [18]. Additionally, when complications arise from IE, surgical intervention is regarded as the optimal management strategy [15].

This review focuses on the diagnostic challenges of very early and early onset IE following TAVI, moving beyond a mere outline of diagnostic and therapeutic strategies. It emphasizes the critical role of specific imaging modalities in detecting IE in patients who have recently undergone TAVI, while addressing the current limitations inherent with these approaches.

## 2. Diagnostic Criteria for IE and Clinical Features of IE Post-TAVI

IE is primarily diagnosed based on the modified major and minor Duke criteria. The major criteria include positive blood cultures and evidence of endocardial involvement with echocardiography; meanwhile, the minor criteria encompass predisposing heart conditions or injection drug use, fever with a temperature >38 °C, vascular phenomena, immunologic phenomena, and microbiological evidence [19]. For prosthetic valve IE, the diagnosis follows these criteria, with a greater emphasis on using TEE due to the superior ability of this method to visualize prosthetic valves and associated complications, such as paravalvular abscesses or dehiscence [19]. Specifically, detecting small vegetation in post-TAVI IE is limited due to the large amount of metal in the valve, which causes reflectance and shadowing effects [20]. Therefore, the 2023 European guidelines have incorporated various imaging techniques into the diagnostic criteria, offering better imaging investigation to diagnose PVE accurately and, subsequently, IE post-TAVI [15].

Patients with IE post-TAVI may exhibit vague complaints and nonspecific symptoms, leading to delays in diagnosis and appropriate management [21]. Fever (71.7%) and heart failure (58.5%), along with nonspecific symptoms such as malaise and fatigue, are among the most common symptoms of IE post-TAVI [22, 23]. Specific symptoms resulting from septic emboli include neurological symptoms; meanwhile, the less commonly observed symptoms include cutaneous stigmata, local chills, loss of appetite, macrophage activation syndrome, and limb ischemia due to septic emboli [23]. Although the modified Duke criteria demonstrate high sensitivity for diagnosing native valve endocarditis (NVE), the sensitivity of these criteria is reduced in cases of PVE [24]. In a comparative study of Duke criteria and those of the ESC, which incorporate multimodal imaging, the Duke criteria exhibited a sensitivity of only 50%. In contrast, the modified ESC criteria exhibited a sensitivity of 100% [25]. A summary of the modified ESC criteria can be found in Appendix Table A1, adapted from Delgado *et al*. [15].

## 3. Risk Factors of IE Post-TAVI

TAVI entails an elevated risk of IE. The incidence of IE post-TAVI ranges from 0.3 to 1.9 per 100 patient–years, with one recent study reporting a lower incidence of IE post-TAVI at approximately 3–10 per 1000 patient–years [15, 26]. This risk is particularly elevated in the first year post-procedure, with a notable increase within the initial 3 months [27, 28]. Both patient-related and procedural-related risk factors significantly contribute to developing IE following TAVI [11].

Patient-related factors, such as younger age, male sex, and comorbidities, including diabetes mellitus, chronic obstructive pulmonary disease, chronic kidney disease, peripheral arterial disease, and an elevated body mass index, all contribute to increased risk [29, 30]. Indeed, a recent meta-analysis comprising over 68,000 patients revealed that older age was linked to a significantly reduced risk of IE post-TAVI [31]. One proposed explanation is that younger patients undergoing TAVI exhibit more pronounced comorbidities compared to their older counterparts [28]. Meanwhile, the lower occurrence of IE post-TAVI in females has been attributed to the protective effects of estrogen on the vascular endothelium [32]. However, further studies are needed to examine the underlying mechanisms thoroughly. While most studies identify male sex as a risk factor for IE post-TAVI, a recent retrospective cohort study showed a similar occurrence between the two genders [29].

Procedural-related factors, such as orotracheal or nasotracheal intubation, heighten the risk of IE post-TAVI due to potential bacteremia following intubation [33]. Additional procedural-related risk factors include residual aortic regurgitation, significant paravalvular leak, and high-degree atrioventricular block post-TAVI, necessitating pacemaker implantation [14, 24, 34].

It is also important to thoroughly discuss the risk factors specific to very early IE post-TAVI since this clinical entity is more challenging to diagnose early, and limited literature is available on the subject. A recent retrospective observational study suggests that female gender is a risk factor, potentially because women undergoing TAVI are often older, frailer, and more immunosuppressed [35, 36, 37, 38]. Furthermore, self-expanding valves have been identified as a risk factor, likely due to their larger stent frame and the fact that their delivery systems often come into direct contact with the skin before valve deployment. This contrasts with balloon-expandable valves, which are always inserted within an external introducer sheath. Sepsis remains a risk factor as a periprocedural complication. Additionally, the occurrence of stroke may increase risk, as this condition requires extensive nursing care and leads to longer hospital stays, thereby heightening exposure to potential pathogens [38].

## 4. Mortality Rates

Recent studies have shown that mortality rates for IE post-TAVI are higher compared with NVE and SAVR-IE. More specifically, 1-year mortality rates for IE post-TAVI range from 40% to 45.6%, while the rates for SAVR-IE range from 23.1% to 32.8%, and for NVE, the rates are approximately 15% to 30% [39, 40, 41, 42, 43]. It is important to highlight that very early IE is associated with the highest mortality rate among patients with IE post-TAVI [38]. However, the mortality rate of IE can also be influenced by microorganisms, with certain organisms being associated with higher mortality rates compared to others [44, 45].

## 5. Microorganisms

Indeed, Gram-positive bacteria are frequently implicated in most reports detailing IE post-TAVI [46]. According to a recent systematic review and meta-analysis, *Streptococcus spp.* (25.3%), *Staphylococcus spp.* (25.3%), and *Enterococcus spp*. (24.1%) were the most commonly identified pathogens causing IE after TAVI [47]. Notably, *Staphylococcus aureus* and *Enterococcus faecalis* were the dominant species, comprising 60% and 65.8% of cases, respectively [48]. Interestingly, *Staphylococcus aureus*, most commonly methicillin-resistant, represented the primary pathogen in very early IE post-TAVI, followed by enterococci organisms as the second most common pathogens [38]. The predominance of *Staphylococcus aureus* in very early IE post-TAVI is due to its natural colonization of the skin, its role as a healthcare-associated pathogen, procedural contamination risks, inadequate prophylactic antibiotic coverage, and its biofilm-forming ability on prosthetic surfaces. Therefore, addressing this issue may involve improving perioperative aseptic protocols, re-evaluating antibiotic prophylaxis to include coverage for MRSA, and adopting strategies to minimize procedural contamination [38].

## 6. Imaging Techniques 

Before we thoroughly analyze the imaging techniques, it is worth mentioning that transcatheter valve prostheses contain leaflets with a higher metal quantity in the stent frame compared to surgical valves [32]. However, no vegetation may be detected in 38–60% of cases [14]. Vegetations are typically located in the stent frame of the transcatheter valve, rather than on the valve leaflets, in 12% of cases. This rate increases to 19% in the presence of some self-expanding valve systems with a longer stent frame that occupies the ascending aorta [49]. Additionally, vegetations are found outside the transcatheter valve in about one-third of cases, primarily at the mitral valve level [14]. Table 1 summarizes the imaging techniques, their indications, strengths, and challenges for diagnosing infective endocarditis post-TAVI.

**Table 1.** **Imaging techniques: indications, strengths, and challenges**.

Imaging technique	Indications	Strengths	Challenges
TTE/TOE	Primary imaging modality for diagnosing IE post-TAVI.	∙ TEE is superior to TTE for detecting vegetation and complications	∙ Artifacts from the prosthetic stent
		∙ TEE offers higher sensitivity and specificity for PVE	∙ Differentiation of vegetation *vs*. fibrous stand *vs*. thrombus
Cardiac CT	Assessing complications, such as abscesses or fistulas, in IE post-TAVI.	∙ Superior for detecting perivalvular/periprosthetic complications	∙ Limited use in detecting complications
		∙ High anatomical detail	
CTA	Evaluation of vegetations, mycotic aneurysms, abscesses, and paravalvular leakage.	∙ Comprehensive imaging of both vascular and valve anatomy	∙ Artifacts from the prosthetic stent
		∙ Non-invasive	∙ Differentiation of vegetation *vs*. thrombus
^18^F-FDG PET/CT(A)	Detecting early-stage infection, monitoring metabolic activity in IE post-TAVI.	∙ High sensitivity	∙ Not reliable the first month after TAVI
		∙ Can detect metabolic changes before structural damage	
		∙ Effective in identifying infection at 1 month post-TAVI	
(99mTc-HMPAO) SPECT/CT	When FDG-PET/CT is unavailable or unreliable due to early post-operative inflammatory changes.	∙ High specificity for early-stage infection	∙ There is no clear reliability in the very early post-TAVI period
	Superior specificity in diagnosing PVE compared to ^18^F-FDG PET/CT.	∙ Can distinguish between normal post-surgical inflammation and infection	∙ Accumulation may be diminished in drained or encapsulated abscesses
MRI	Assessing valve function and detecting abscesses or thrombus post-TAVI.	∙ Excellent soft tissue contrast	∙ Lower spatial resolution
		∙ Good for monitoring myocardial involvement	∙ Signal voids
ICE	Detection of early or very early-stage vegetations and complications post-TAVI.	∙ High-resolution imaging of prosthetic valve structures	∙ Invasive procedure
		∙ Direct visualization of vegetation	∙ Not widely used

99mTc-HMPAO, technetium-99m-hexamethylpropyleneamineoxime-labeled leukocytes; MRI, magnetic resonance imaging; ICE, intracardiac echocardiography; ^18^F-FDG PET/CT, positron emission tomography/computed tomography using 18F-fluorodeoxyglucose; TAVI, transcatheter aortic valve implantation; TTE, transthoracic echocardiography; TOE, transesophageal echocardiography; TEE, transesophageal echocardiography; PVE, prosthetic valve endocarditis; IE, infective endocarditis; CTA, computed tomography angiography; SPECT, single photon emission computed tomography.

### 6.1 Transthoracic/Transesophageal Echocardiography 

**Fig. 1.**
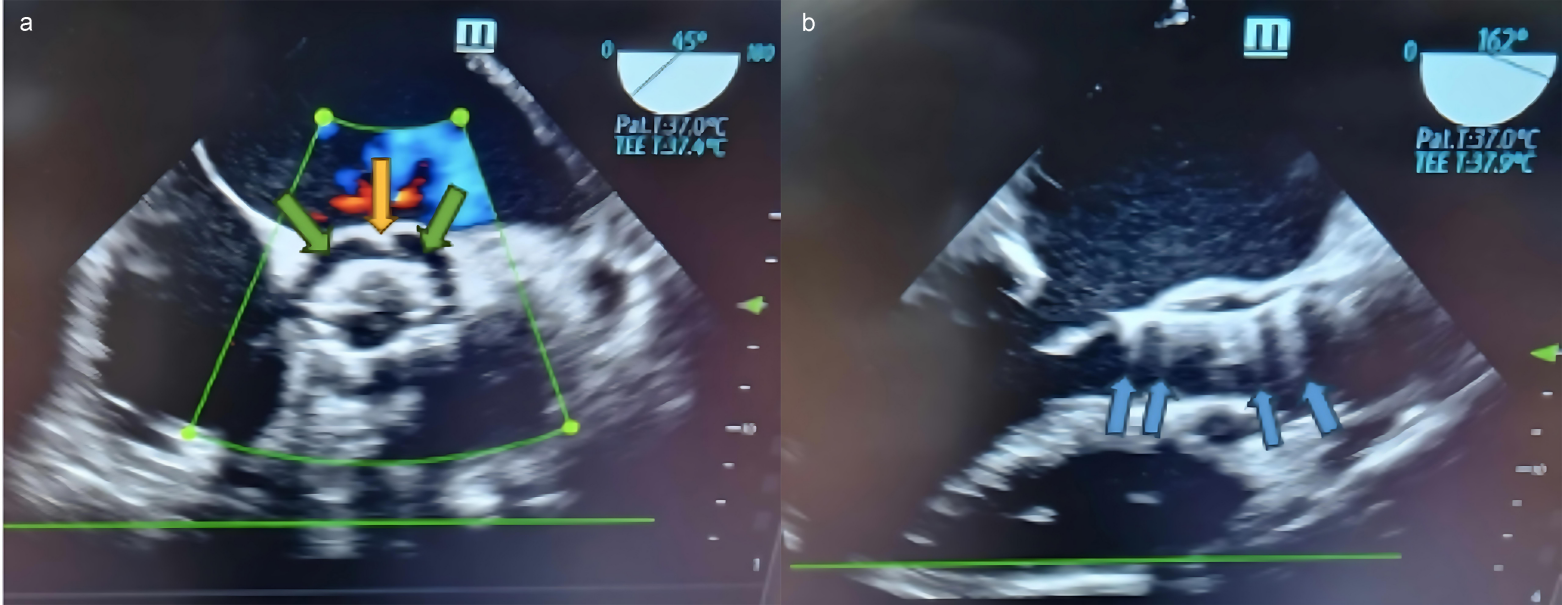
**Infective endocarditis of a prosthetic aortic valve**. (a) Mid-esophageal aortic valve short-axis view of vegetation at the 12 o’clock position (yellow arrow) in the stent frame of the transcatheter valve, along with a paravalvular leak (green arrows). (b) Mid-esophageal aortic valve long-axis view. Same patient. Blue arrows show the shadowing effect and the reflectance of the prosthetic valve. TEE, transesophageal echocardiography.

Echocardiography serves as the primary imaging modality for diagnosing IE post-TAVI, regardless of the timing of the disease, whether it is very early, early, intermediate, or late. Meanwhile, for NVE, TTE demonstrates a sensitivity of approximately 71% and a specificity of 80% [50]. In contrast, TEE offers superior diagnostic accuracy, with sensitivities ranging from 87% to 100% and specificities between 91% and 100% [51, 52, 53]. However, in cases of PVE, the sensitivity of TTE diminishes to 36–69%, while TEE maintains a higher sensitivity, reported between 86% and 100% [51, 54]. However, using TTE and TEE to detect small vegetations or paravalvular abscesses is challenging due to the shadowing effect and reflectance of prosthesis material. Moreover, detecting incipient vegetation in the free space between the transcatheter and native aortic valve, amidst extensive calcifications and prosthetic material, is particularly challenging using TTE and TEE, if even possible [55]. TTE and TEE may also be unable to differentiate between vegetations and fibrous strands or thrombi [28]. Fig. 1a,b illustrates the concepts mentioned above.

Despite these challenges, while TTE is recommended as the initial imaging procedure for all patients suspected of having NVE or PVE, it does not hold the status of the gold standard [56]. In all cases of alleged PVE, TEE remains essential for valvular hemodynamics assessment and the potential detection of vegetation, abscess, or fistula. To conclude, TEE is strongly recommended in patients with an inconclusive TTE, in those with a negative TTE and a high suspicion of IE, in all patients with clinical suspicion when a prosthetic valve or an intracardiac device is present, as well as in patients with a positive TTE to document local complications [15]. It is worth mentioning that echocardiography is necessary in any case of bacteremia with *Staphylococcus aureus* [15]. Moreover, the echocardiographic examination must be repeated after five to seven days in case of unconfirmed but highly suggestive PVE [56].

### 6.2 Cardiac Computed Tomography and Computed Tomography Angiography

Cardiac CT is another imaging modality that can assist in diagnosing IE following TAVI, regardless of the timing of the disease, by providing superior visualization of its complications. Cardiac CT is superior to TEE in accurately diagnosing perivalvular and periprosthetic complications of IE. Moreover, cardiac CT may be useful in identifying these crucial complications in cases where a paravalvular abscess, pseudoaneurysm, or fistulae is suspected with equivocal findings from TEE [57, 58]. Nonetheless, TEE remains the gold standard for detecting valvular lesions, particularly small vegetations (<10 mm) [58]. Additionally, it is important to note that TEE demonstrates superior accuracy compared to cardiac CT in detecting leaflet perforations [59].

CTA can be utilized as a diagnostic imaging technique for assessing IE post-TAVI, irrespective of the timing of onset, enabling the visualization of features such as vegetations, mycotic aneurysms, abscesses, paravalvular leakage, and valve dehiscence. However, recognizing the inherent challenges when interpreting valvular abnormalities on CTA remains crucial since artifacts from prosthetic stent materials may pose challenges in accurately assessing valvular irregularities, potentially resulting in false-negative outcomes. Furthermore, post-TAVI leaflet thrombosis, also known as hypoattenuating leaflet thickening (HALT), may occur even in patients receiving anticoagulation therapy, adding another layer of complexity to the interpretation. Indeed, the possibility of misinterpreting HALT as vegetation must be considered, as it can result in false-positive CTA findings [60].

It is also important to acknowledge that contrast-induced nephropathy is typically reversible, often presenting as a mild reduction in the glomerular filtration rate (GFR), which improves within three to seven days; most patients return to or near their baseline estimated GFR. However, individuals with advanced renal failure may require temporary dialysis following contrast administration [61]. Therefore, performing CTA can contribute to the diagnosis in cases where echocardiography provides equivocal findings.

### 6.3 Nuclear Imaging: ^18^F-FDG PET/CT

^18^F-FDG PET/CT was incorporated into the ESC modified criteria to enhance sensitivity [15]. The most recent meta-analysis found that this imaging method had a sensitivity of 86% [62]. Thus, abnormal FDG uptake can be observed before the manifestation of infectious damage in echocardiography by assessing metabolic tissue activity. This underscores the capability of ^18^F-FDG PET/CT to detect infection before significant damage has occurred [63]. Increased metabolic activity in the heart can mimic the patterns seen in IE due to the inflammatory response following TAVI. This heightened metabolic activity can potentially lead to false-positive interpretations on ^18^F-FDG PET/CT scans [63]. While the ESC guidelines recommend performing ^18^F-FDG PET/CT three months after cardiac surgery to reduce the risk of false-positive results related to the post-operative inflammatory process [64], limited data exist for very early IE post-TAVI. Indeed, recent data suggest that ^18^F-FDG PET/CT can be accurately used at least one month after TAVI to diagnose IE post-TAVI, owing to the distinct FDG uptake patterns observed during this period. Specifically, circumferential or hemi-circumferential uptake represents a normal post-TAVI pattern, whereas focal or multifocal uptake strongly suggests definite IE–TAVI, enhancing diagnostic specificity [65].

Furthermore, ^18^F-FDG PET/CT imaging presents technical challenges, including adequate patient preparation. This typically involves fasting at least 6 hours before the procedure and heparin administration to minimize false-positive results and optimize imaging quality [66]. Additionally, it is crucial to ensure that the patient has proper blood glucose control, as elevated levels can interfere with the accuracy of the FDG uptake assessment. Moreover, PET/CT has limitations in detecting small foci during inflammation, which may result in false-negative findings, particularly in early-stage infections. Studies have also highlighted that PET imaging may not be as effective in detecting localized infection in patients with low-grade inflammatory responses [67].

Combining PET/CT acquisition with CT angiography (PET/CTA) enables the detection of both metabolic findings (such as ^18^F-FDG uptake distribution and intensity) and anatomical findings (related to infective endocarditis lesions) within a single imaging procedure [60, 68].

### 6.4 White Blood Cell SPECT/CT

WBC SPECT/CT is an alternative nuclear imaging technique for diagnosing IE, when PET/CT is unavailable and in inexperienced centers, with a sensitivity of 64–90% [15]. WBC SPECT/CT, specifically using technetium-99m-hexamethylpropyleneamineoxime-labeled leukocytes (99mTc-HMPAO), is a highly specific imaging modality for diagnosing infections, including IE, even within the first month after TAVI. Moreover, WBC SPECT/CT can effectively differentiate between normal post-surgical inflammatory uptake and abnormal patterns indicative of infection. Studies have demonstrated its specificity—up to 100%—in early post-operative settings when other imaging methods, such as ^18^F-FDG PET/CT, may be less reliable due to nonspecific inflammatory changes. WBC SPECT/CT can be used to diagnose both intracardiac and extracardiac infections [69].

Thus, WBC SPECT/CT has emerged as a valuable tool in reducing the number of misdiagnosed cases of IE, previously classified as “possible IE” by the modified Duke criteria, with a significant decrease of 27% [70]. This imaging technique demonstrates high specificity in identifying active infective processes in perivalvular regions, and, as mentioned, shows superior specificity in diagnosing PVE compared to ^18^F-FDG PET [71]. However, it is essential to recognize that WBC accumulation may be diminished in certain conditions, such as in drained or encapsulated abscesses or non-pyogenic bacterial infections. This highlights the importance of using WBC SPECT/CT in the appropriate clinical context to maximize diagnostic accuracy [72].

### 6.5 Magnetic Resonance Imaging

While MRI is a common modality in diagnosing stroke and embolic events, its role in diagnosing IE is limited [15]. Especially in PVE, the value of MRI is low due to artifact interference. Nevertheless, in cases with high clinical suspicion of IE, cardiac MRI can provide valuable diagnostic and prognostic information by depicting features such as antegrade and retrograde dissemination, paravalvular tissue extension, and subendocardial and vascular endothelial involvement for delayed contrast-enhanced images [73]. Despite its potential, cardiac MRI is not a replacement for echocardiography but a complementary tool in specific scenarios. Additionally, the diagnostic potential of MRI is constrained by a lower spatial resolution than cardiac CT, and signal voids from certain prostheses may hinder the precise evaluation of the prosthetic valve anatomy and function [74].

### 6.6 Intracardiac Echocardiography

ICE is a valuable tool for diagnosing IE post-TAVI, particularly in the early and very early stages. ICE presents a distinct advantage by directly visualizing prosthetic valve leaflets on the endocardial surface, overcoming challenges posed by acoustic shadowing from metallic stent frames. While TTE and TEE are commonly used, ICE provides real-time, high-resolution imaging, offering greater sensitivity and specificity when evaluating valvular and perivalvular structures [75]. In cases of early IE post-TAVI, ICE is particularly useful for identifying vegetations, abscesses, and complications such as valve perforations or pseudoaneurysms. Vegetations, a hallmark of IE, appear as oscillating or non-oscillating masses on the valve leaflets and are often better visualized using TEE or ICE compared to TTE, especially in the context of complex intracardiac structures, such as prosthetic valves [75].

Despite this, the major drawback lies in the invasive nature of the procedure. Typically performed via venous access to mitigate vascular complications, ICE becomes more complex in aortic valve prosthesis endocarditis, necessitating arterial access and elevating the risk of vascular complications. Additionally, the retrograde introduction of the ICE catheter across the aortic arch introduces potential risks such as aortic wall injury, dissection, or stroke. While theoretically possible, there have been no reported instances of embolization of vegetation by the ICE catheter, despite its proximity to the infected valve endocardial surface [76, 77].

In summary, ICE offers superior imaging for detecting and assessing the complications of IE early in the disease process. Moreover, ICE allows clinicians to accurately monitor patients and intervene promptly when other imaging modalities are limited.

### 6.7 Detection of Distant Lesions

Identifying distant lesions represents a minor diagnostic criterion, crucial for achieving a more conclusive, definite, or rejected IE diagnosis, and plays a significant role in guiding treatment decisions. Whole-body and brain CT scans offer valuable insights into systemic complications of IE, including septic emboli [78, 79]. MRI, boasting higher sensitivity than CT, excels in detecting neurological lesions, thereby improving the diagnosis of neurological complications in IE patients [79]. Moreover, whole-body ^18^F-FDG PET/CT imaging proves invaluable in suspected or confirmed IE cases, facilitating the identification of distant lesions and mycotic aneurysms and pinpointing the site of infection entry [60, 80].

**Fig. 2.**
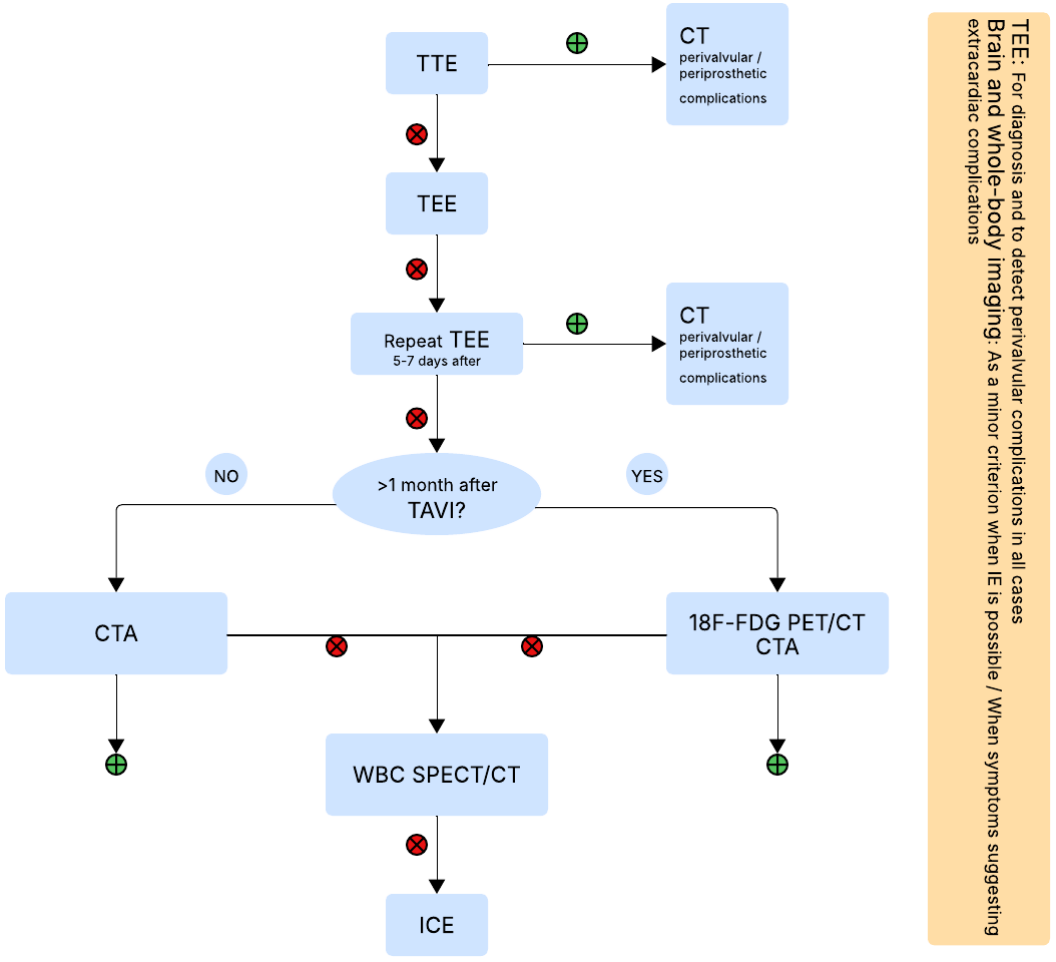
**Proposed algorithm for diagnosis of IE post-TAVI**. CT, computed tomography; ^18^F-FDG, ^18^F-fluorodeoxyglucose; PET, positron emission tomography; WBC SPECT, white blood cell single photon emission tomography; TTE, transthoracic echocardiography; TEE, transesophageal echocardiography; IE, infective endocarditis; TAVI, transcatheter aortic valve implantation; ICE, intracardiac echocardiography; CTA, computed tomography angiography. Fig. 2 was created using draw.io.

Based on the aforementioned imaging techniques, a proposed algorithm for their use in diagnosing IE post-TAVI is presented in Fig. 2.

## 7. Treatment

The treatment approach for IE is multifaceted, involving both antimicrobial therapy and, in many cases, surgical intervention.

In suspected cases of IE, empirical antibiotic therapy should be initiated promptly, even before blood culture results are available, to cover the most likely pathogens. Considering the microbiological profile, initial empirical treatment should encompass effective agents against methicillin-resistant staphylococci and enterococci. Subsequently, therapy should be tailored to the identified pathogens once blood cultures and sensitivities are available. The PVE antibiotic treatment duration should last longer (≥6 weeks) than that of NVE (2–6 weeks), but is otherwise similar [15].

Surgery is necessary when complications such as heart failure, uncontrolled infection, established embolism, or a high risk of embolism are present. The timing of surgical intervention is crucial and should be individualized based on the clinical condition of each patient: emergency surgery within 24 hours, urgent surgery within 3–5 days, and non-urgent surgery within the same hospital admission. The decision to proceed with surgery in IE post-TAVI patients should be individualized, balancing the surgical risks and the prognosis of medical treatment alone [15]. Despite notably high in-hospital mortality rates, very few individuals with indications for surgery undergo the procedure. This reluctance may be attributed to the significant surgical risks associated with this older population, which often presents with multiple comorbidities [38]. Nevertheless, current evidence suggests that surgical treatment may provide up to a 20% survival advantage in the first year [81].

## 8. Conclusion

Diagnosing IE following TAVI presents challenges. The complexity of diagnosing infective endocarditis post-TAVI increases significantly when the condition manifests early after the TAVI procedure (0–60 days). Despite advanced imaging techniques and their diagnostic sensitivity, a diagnosis of IE in the initial diagnostic examination may remain possible rather than certain. This is due to falsely negative imaging findings presented by each modality, as well as the limited availability or use of certain imaging techniques.

This review addresses critical gaps in the diagnostic landscape of TAVI-related IE, focusing on the very early and early onset of this complication. While previous studies have primarily centered on clinical outcomes and management strategies, our work uniquely emphasizes the diagnostic challenges associated with the early detection of IE [82]. By highlighting the limitations of current imaging modalities, we advocate for a multimodal imaging approach that synthesizes recent advancements in the field. This perspective enriches the understanding of TAVI-related IE and offers practical insights for clinicians. Future research should continue to explore innovative imaging techniques and their integration into clinical practice to enhance early detection and improve patient outcomes in this rapidly evolving area.

In conclusion, early detection of IE following TAVI is vital, as delayed diagnosis can dramatically worsen patient outcomes. Although imaging techniques play a central role, their sensitivity is limited, particularly in the early stages of the disease. Therefore, maintaining a high index of clinical suspicion, prompting microbiological testing, and initiating broad-spectrum empirical antibiotic therapy, including coverage for MRSA, is essential for effective management. Enhancing diagnostic approaches and refining treatment protocols are critical steps toward reducing mortality and improving recovery rates in this high-risk patient population.

## Appendix

See Appendix Table A1.

**Table A1.** **The modified European Society of Cardiology diagnostic criteria for infective endocarditis**.

Major criteria		
	1. Blood cultures positive for IE:	
		A. Typical microorganisms consistent with IE from two separate blood cultures:
		∙ Oral streptococci, *Streptococcus gallolyticus* (formerly *S. bovis*), HACEK group, *S. aureus*, *E*. *faecalis*.
		B. Microorganisms consistent with IE from continuously positive blood cultures:
		∙ ≥2 positive blood cultures of blood samples drawn >12 h apart.
		∙ All 3 or mostly ≥4 separate blood cultures (with first and last samples drawn ≥1 h apart).
		C. Single positive blood culture for *C. burnetii* or phase I IgG antibody titer >1:800.
	2. Imaging positive for IE:	
		∙ Valvular, perivalvular/periprosthetic, and foreign material anatomic and metabolic lesions characteristic of IE detected using any of the following imaging techniques:
		∙ Echocardiography (TTE and TOE).
		∙ Cardiac CT.
		∙^18^F-FDG-PET/CT(A).
		∙ WBC SPECT/CT.
Minor criteria		
	1. Predisposing conditions (i.e., predisposing heart condition at high or intermediate risk of IE or PWIDs).	
	2. Fevers with a temperature >38 °C.	
	3. Embolic vascular dissemination (including those asymptomatic detected by imaging only):	
		∙ Major systemic and pulmonary emboli/infarcts and abscesses.
		∙ Hematogenous osteoarticular septic complications (i.e., spondylodiscitis).
		∙ Mycotic aneurysms.
		∙ Intracranial ischemic/hemorrhagic lesions.
		∙ Conjunctival hemorrhages.
		∙ Janeway’s lesions.
	4. Immunological phenomena:	
		∙ Glomerulonephritis.
		∙ Osler nodes and Roth spots.
		∙ Rheumatoid factor.
	5. Microbiological evidence:	
		∙ Positive blood culture; however, none of the major criteria noted above are met.
		∙ Serological evidence of active infection with organism consistent with IE.
IE classification		
Definite ΙΕ:		
	2 major criteria, or	
	1 major criterion and at least 3 minor criteria, or	
	5 minor criteria.	
Possible ΙΕ:		
	1 major criterion and 1 or 2 minor criteria, or	
	3–4 minor criteria.	
Rejected ΙΕ:		
	None of the criteria for definite or possible are met at admission with or without a firm alternative diagnosis.	

^18^F-FDG-PET/CT, positron emission tomography/computed tomography using ^18^F-fluorodeoxyglucose; CT(A), computed tomography (angiography); HACEK, *Haemophilus*, *Aggregatibacter*, *Cardiobacterium*, *Eikenella*, and *Kingella*; IE, infective endocarditis; Ig, immunoglobulin; PWIDs, people who inject drugs; TOE, transesophageal echocardiography; TTE, transthoracic echocardiography; WBC SPECT/CT, white blood cell single-photon emission computed tomography/computed tomography.

## References

[1] Vahanian A, Beyersdorf F, Praz F, Milojevic M, Baldus S, Bauersachs J (2022). 2021 ESC/EACTS Guidelines for the management of valvular heart disease: developed by the Task Force for the management of valvular heart disease of the European Society of Cardiology (ESC) and the European Association for Cardio-Thoracic Surgery (EACTS). *European Heart Journal*.

[2] Williams MR, Jilaihawi H, Makkar R, O’Neill WW, Guyton R, Malaisrie SC (2022). The PARTNER 3 Bicuspid Registry for Transcatheter Aortic Valve Replacement in Low-Surgical-Risk Patients. *Cardiovascular Interventions*.

[3] Waksman R, Craig PE, Torguson R, Asch FM, Weissman G, Ruiz D (2020). Transcatheter Aortic Valve Replacement in Low-Risk Patients With Symptomatic Severe Bicuspid Aortic Valve Stenosis. *Cardiovascular Interventions*.

[4] Forrest JK, Ramlawi B, Deeb GM, Zahr F, Song HK, Kleiman NS (2021). Transcatheter Aortic Valve Replacement in Low-risk Patients With Bicuspid Aortic Valve Stenosis. *JAMA Cardiology*.

[5] Spitzer E, Van Mieghem NM, Pibarot P, Hahn RT, Kodali S, Maurer MS (2016). Rationale and design of the Transcatheter Aortic Valve Replacement to UNload the Left ventricle in patients with ADvanced heart failure (TAVR UNLOAD) trial. *American Heart Journal*.

[6] Ludwig S, Schofer N, Abdel-Wahab M, Urena M, Jean G, Renker M (2023). Transcatheter Aortic Valve Replacement in Patients With Reduced Ejection Fraction and Nonsevere Aortic Stenosis. *Circulation. Cardiovascular Interventions*.

[7] Lu L, Sun R, Liu M, Zheng Y, Zhang P (2015). The Inflammatory Heart Diseases: Causes, Symptoms, and Treatments. *Cell Biochemistry and Biophysics*.

[8] Werdan K, Dietz S, Löffler B, Niemann S, Bushnaq H, Silber RE (2014). Mechanisms of infective endocarditis: pathogen-host interaction and risk states. *Nature Reviews. Cardiology*.

[9] Mack MJ, Leon MB, Thourani VH, Makkar R, Kodali SK, Russo M (2019). Transcatheter Aortic-Valve Replacement with a Balloon-Expandable Valve in Low-Risk Patients. *The New England Journal of Medicine*.

[10] Olsen NT, De Backer O, Thyregod HGH, Vejlstrup N, Bundgaard H, Søndergaard L (2015). Prosthetic valve endocarditis after transcatheter aortic valve implantation. *Circulation. Cardiovascular Interventions*.

[11] Del Val D, Panagides V, Mestres CA, Miró JM, Rodés-Cabau J (2023). Infective Endocarditis After Transcatheter Aortic Valve Replacement: JACC State-of-the-Art Review. *Journal of the American College of Cardiology*.

[12] Østergaard L, Lauridsen TK, Iversen K, Bundgaard H, Søndergaard L, Ihlemann N (2020). Infective endocarditis in patients who have undergone transcatheter aortic valve implantation: a review. *Clinical Microbiology and Infection: the Official Publication of the European Society of Clinical Microbiology and Infectious Diseases*.

[13] Panagides V, Del Val D, Abdel-Wahab M, Mangner N, Durand E, Ihlemann N (2022). Perivalvular Extension of Infective Endocarditis After Transcatheter Aortic Valve Replacement. *Clinical Infectious Diseases: an Official Publication of the Infectious Diseases Society of America*.

[14] Bjursten H, Rasmussen M, Nozohoor S, Götberg M, Olaison L, Rück A (2019). Infective endocarditis after transcatheter aortic valve implantation: a nationwide study. *European Heart Journal*.

[15] Delgado V, Ajmone Marsan N, de Waha S, Bonaros N, Brida M, Burri H (2023). 2023 ESC guidelines for the management of endocarditis: developed by the task force on the management of endocarditis of the European Society of Cardiology (ESC) endorsed by the European Association for Cardio-Thoracic Surgery (EACTS) and the European Association of Nuclear Medicine (EANM). *European Heart Journal*.

[16] Fowler VG, Durack DT, Selton-Suty C, Athan E, Bayer AS, Chamis AL (2023). The 2023 Duke-International Society for Cardiovascular Infectious Diseases Criteria for Infective Endocarditis: Updating the Modified Duke Criteria. *Clinical infectious diseases: an official publication of the Infectious Diseases Society of America*.

[17] Moriyama N, Laakso T, Biancari F, Raivio P, Jalava MP, Jaakkola J (2019). Prosthetic valve endocarditis after transcatheter or surgical aortic valve replacement with a bioprosthesis: results from the FinnValve Registry. *EuroIntervention: Journal of EuroPCR in Collaboration with the Working Group on Interventional Cardiology of the European Society of Cardiology*.

[18] Thuny F, Grisoli D, Cautela J, Riberi A, Raoult D, Habib G (2014). Infective endocarditis: prevention, diagnosis, and management. *The Canadian Journal of Cardiology*.

[19] Li JS, Sexton DJ, Mick N, Nettles R, Fowler VG Jr, Ryan T (2000). Proposed modifications to the Duke criteria for the diagnosis of infective endocarditis. *Clinical Infectious Diseases: An Official Publication of the Infectious Diseases Society of America*.

[20] Habib G (2018). Infective Endocarditis After Transcatheter Aortic Valve Replacement: The Worst That Can Happen. *Journal of the American Heart Association*.

[21] Loh PH, Bundgaard HS, Ndergaard L (2013). Infective endocarditis following transcatheter aortic valve replacement: diagnostic and management challenges. *Catheterization and Cardiovascular Interventions: Official Journal of the Society for Cardiac Angiography & Interventions*.

[22] Chourdakis E, Koniari I, Hahalis G, Kounis NG, Hauptmann KE (2018). Endocarditis after transcatheter aortic valve implantation: a current assessment. *Journal of Geriatric Cardiology: JGC*.

[23] Amat-Santos IJ, Ribeiro HB, Urena M, Allende R, Houde C, Bédard E (2015). Prosthetic valve endocarditis after transcatheter valve replacement: a systematic review. *JACC. Cardiovascular Interventions*.

[24] Kuttamperoor F, Yandrapalli S, Siddhamsetti S, Frishman WH, Tang GHL (2019). Infectious Endocarditis After Transcatheter Aortic Valve Replacement: Epidemiology and Outcomes. *Cardiology in Review*.

[25] Salaun E, Sportouch L, Barral PA, Hubert S, Lavoute C, Casalta AC (2018). Diagnosis of Infective Endocarditis After TAVR: Value of a Multimodality Imaging Approach. *JACC. Cardiovascular Imaging*.

[26] Cahill TJ, Raby J, Jewell PD, Brennan PF, Banning AP, Byrne J (2022). Risk of infective endocarditis after surgical and transcatheter aortic valve replacement. *Heart (British Cardiac Society)*.

[27] Del Val D, Abdel-Wahab M, Linke A, Durand E, Ihlemann N, Urena M (2021). Temporal Trends, Characteristics, and Outcomes of Infective Endocarditis After Transcatheter Aortic Valve Replacement. *Clinical Infectious Diseases: an Official Publication of the Infectious Diseases Society of America*.

[28] Zakhour J, Allaw F, Kalash S, Wehbe S, Kanj SS (2023). Infective Endocarditis after Transcatheter Aortic Valve Replacement: Challenges in the Diagnosis and Management. *Pathogens (Basel, Switzerland)*.

[29] Panagides V, Abdel-Wahab M, Mangner N, Durand E, Ihlemann N, Urena M (2022). Sex Differences in Infective Endocarditis After Transcatheter Aortic Valve Replacement. *The Canadian Journal of Cardiology*.

[30] Mangner N, Woitek F, Haussig S, Schlotter F, Stachel G, Höllriegel R (2016). Incidence, Predictors, and Outcome of Patients Developing Infective Endocarditis Following Transfemoral Transcatheter Aortic Valve Replacement. *Journal of the American College of Cardiology*.

[31] Jiang W, Wu W, Guo R, Xie M, Yim WY, Wang Y (2021). Predictors of Prosthetic Valve Endocarditis following Transcatheter Aortic Valve Replacement: A Meta-Analysis. *The Heart Surgery Forum*.

[32] Amat-Santos IJ, Messika-Zeitoun D, Eltchaninoff H, Kapadia S, Lerakis S, Cheema AN (2015). Infective endocarditis after transcatheter aortic valve implantation: results from a large multicenter registry. *Circulation*.

[33] Valdés C, Tomás I, Alvarez M, Limeres J, Medina J, Diz P (2008). The incidence of bacteraemia associated with tracheal intubation. *Anaesthesia*.

[34] Smolka G, Wojakowski W (2010). Paravalvular leak-important complication after implantation of prosthetic valve. *E-journal of Cardiology Practice*.

[35] Chandrasekhar J, Dangas G, Yu J, Vemulapalli S, Suchindran S, Vora AN (2016). Sex-Based Differences in Outcomes With Transcatheter Aortic Valve Therapy: TVT Registry From 2011 to 2014. *Journal of the American College of Cardiology*.

[36] Sambola A, Fernández-Hidalgo N, Almirante B, Roca I, González-Alujas T, Serra B (2010). Sex differences in native-valve infective endocarditis in a single tertiary-care hospital. *The American Journal of Cardiology*.

[37] Aksoy O, Meyer LT, Cabell CH, Kourany WM, Pappas PA, Sexton DJ (2007). Gender differences in infective endocarditis: pre- and co-morbid conditions lead to different management and outcomes in female patients. *Scandinavian Journal of Infectious Diseases*.

[38] Panagides V, Abdel-Wahab M, Mangner N, Durand E, Ihlemann N, Urena M (2022). Very early infective endocarditis after transcatheter aortic valve replacement. *Clinical Research in Cardiology: Official Journal of the German Cardiac Society*.

[39] Butt JH, Ihlemann N, De Backer O, Søndergaard L, Havers-Borgersen E, Gislason GH (2019). Long-Term Risk of Infective Endocarditis After Transcatheter Aortic Valve Replacement. *Journal of the American College of Cardiology*.

[40] Mentias A, Girotra S, Desai MY, Horwitz PA, Rossen JD, Saad M (2020). Incidence, Predictors, and Outcomes of Endocarditis After Transcatheter Aortic Valve Replacement in the United States. *JACC. Cardiovascular Interventions*.

[41] Fauchier L, Bisson A, Herbert J, Lacour T, Bourguignon T, Etienne CS (2020). Incidence and outcomes of infective endocarditis after transcatheter aortic valve implantation versus surgical aortic valve replacement. *Clinical Microbiology and Infection: the Official Publication of the European Society of Clinical Microbiology and Infectious Diseases*.

[42] Yilmaz Ak H, Özşahin Y, Yesiltas MA, Haberal İ, Kahraman S, Oksen D (2021). Comparison of Demographic Profile, Laboratory, Epidemiology and Clinical Outcomes in Patients with Native Valve and Prosthetic Valve Endocarditis. *The Heart Surgery Forum*.

[43] De Palo M, Scicchitano P, Malvindi PG, Paparella D (2021). Endocarditis in Patients with Aortic Valve Prosthesis: Comparison between Surgical and Transcatheter Prosthesis. *Antibiotics (Basel, Switzerland)*.

[44] Strange JE, Østergaard L, Køber L, Bundgaard H, Iversen K, Voldstedlund M (2023). Patient Characteristics, Microbiology, and Mortality of Infective Endocarditis After Transcatheter Aortic Valve Implantation. *Clinical Infectious Diseases: an Official Publication of the Infectious Diseases Society of America*.

[45] Shah ASV, McAllister DA, Gallacher P, Astengo F, Rodríguez Pérez JA, Hall J (2020). Incidence, Microbiology, and Outcomes in Patients Hospitalized With Infective Endocarditis. *Circulation*.

[46] Khan A, Aslam A, Satti KN, Ashiq S (2020). Infective endocarditis post-transcatheter aortic valve implantation (TAVI), microbiological profile and clinical outcomes: A systematic review. *PloS One*.

[47] Tinica G, Tarus A, Enache M, Artene B, Rotaru I, Bacusca A (2020). Infective endocarditis after TAVI: a meta-analysis and systematic review of epidemiology, risk factors and clinical consequences. *Reviews in Cardiovascular Medicine*.

[48] Alexis SL, Malik AH, George I, Hahn RT, Khalique OK, Seetharam K (2020). Infective Endocarditis After Surgical and Transcatheter Aortic Valve Replacement: A State of the Art Review. *Journal of the American Heart Association*.

[49] Regueiro A, Linke A, Latib A, Ihlemann N, Urena M, Walther T (2019). Infective Endocarditis Following Transcatheter Aortic Valve Replacement: Comparison of Balloon- Versus Self-Expandable Valves. *Circulation. Cardiovascular Interventions*.

[50] Bonzi M, Cernuschi G, Solbiati M, Giusti G, Montano N, Ceriani E (2018). Diagnostic accuracy of transthoracic echocardiography to identify native valve infective endocarditis: a systematic review and meta-analysis. *Internal and Emergency Medicine*.

[51] Maurice A, Sherman J, Daley N, Collins K, Burstow D, Platts D (2013). The sensitivity and specificity of Modern-Era 2D/3D transoesophageal and transthoracic echocardiography for diagnosis of native and prosthetic valve left-sided infective endocarditis compared with surgical findings. *Heart, Lung and Circulation*.

[52] Silbiger JJ, Rashed E, Chen H, Wiesenfeld E, Robinson SE, Cagliostro M (2022). Cardiac Imaging for Diagnosis and Management of Infective Endocarditis. *Journal of the American Society of Echocardiography: Official Publication of the American Society of Echocardiography*.

[53] Cahill TJ, Baddour LM, Habib G, Hoen B, Salaun E, Pettersson GB (2017). Challenges in Infective Endocarditis. *Journal of the American College of Cardiology*.

[54] Daniel WG, Mügge A, Grote J, Hausmann D, Nikutta P, Laas J (1993). Comparison of transthoracic and transesophageal echocardiography for detection of abnormalities of prosthetic and bioprosthetic valves in the mitral and aortic positions. *The American Journal of Cardiology*.

[55] Puls M, Eiffert H, Hünlich M, Schöndube F, Hasenfuß G, Seipelt R (2013). Prosthetic valve endocarditis after transcatheter aortic valve implantation: the incidence in a single-centre cohort and reflections on clinical, echocardiographic and prognostic features. *EuroIntervention: Journal of EuroPCR in Collaboration with the Working Group on Interventional Cardiology of the European Society of Cardiology*.

[56] Ivanovic B, Trifunovic D, Matic S, Petrovic J, Sacic D, Tadic M (2019). Prosthetic valve endocarditis - A trouble or a challenge?. *Journal of Cardiology*.

[57] Sifaoui I, Oliver L, Tacher V, Fiore A, Lepeule R, Moussafeur A (2020). Diagnostic Performance of Transesophageal Echocardiography and Cardiac Computed Tomography in Infective Endocarditis. *Journal of the American Society of Echocardiography: Official Publication of the American Society of Echocardiography*.

[58] Jain V, Wang TKM, Bansal A, Farwati M, Gad M, Montane B (2021). Diagnostic performance of cardiac computed tomography versus transesophageal echocardiography in infective endocarditis: A contemporary comparative meta-analysis. *Journal of Cardiovascular Computed Tomography*.

[59] Oliveira M, Guittet L, Hamon M, Hamon M (2020). Comparative Value of Cardiac CT and Transesophageal Echocardiography in Infective Endocarditis: A Systematic Review and Meta-Analysis. *Radiology. Cardiothoracic Imaging*.

[60] Wahadat AR, Tanis W, Swart LE, Scholtens A, Krestin GP, van Mieghem NMDA (2021). Added value of 18F-FDG-PET/CT and cardiac CTA in suspected transcatheter aortic valve endocarditis. *Journal of Nuclear Cardiology: Official Publication of the American Society of Nuclear Cardiology*.

[61] Modi K, Padala SA, Gupta M (2025). Contrast-Induced Nephropathy. *In StatPearls*.

[62] Wang TKM, Sánchez-Nadales A, Igbinomwanhia E, Cremer P, Griffin B, Xu B (2020). Diagnosis of Infective Endocarditis by Subtype Using 18F-Fluorodeoxyglucose Positron Emission Tomography/Computed Tomography: A Contemporary Meta-Analysis. *Circulation. Cardiovascular Imaging*.

[63] Saby L, Laas O, Habib G, Cammilleri S, Mancini J, Tessonnier L (2013). Positron emission tomography/computed tomography for diagnosis of prosthetic valve endocarditis: increased valvular 18F-fluorodeoxyglucose uptake as a novel major criterion. *Journal of the American College of Cardiology*.

[64] Habib G, Lancellotti P, Antunes MJ, Bongiorni MG, Casalta JP, Del Zotti F (2015). 2015 ESC Guidelines for the management of infective endocarditis: The Task Force for the Management of Infective Endocarditis of the European Society of Cardiology (ESC). Endorsed by: European Association for Cardio-Thoracic Surgery (EACTS), the European Association of Nuclear Medicine (EANM). *European Heart Journal*.

[65] San S, Abulizi M, Moussafeur A, Oliver L, Lepeule R, Nahory L (2019). Characterization of 18-Fluorodeoxyglucose Uptake Pattern in Infective Endocarditis After Transcatheter Aortic Valve Implantation. *JACC. Cardiovascular Imaging*.

[66] Yan J, Zhang C, Niu Y, Yuan R, Zeng X, Ge X (2016). The role of 18F-FDG PET/CT in infectious endocarditis: a systematic review and meta-analysis. *International Journal of Clinical Pharmacology and Therapeutics*.

[67] Pijl JP, Nienhuis PH, Kwee TC, Glaudemans AWJM, Slart RHJA, Gormsen LC (2021). Limitations and Pitfalls of FDG-PET/CT in Infection and Inflammation. *Seminars in Nuclear Medicine*.

[68] Pizzi MN, Roque A, Fernández-Hidalgo N, Cuéllar-Calabria H, Ferreira-González I, Gonzàlez-Alujas MT (2015). Improving the Diagnosis of Infective Endocarditis in Prosthetic Valves and Intracardiac Devices With 18F-Fluordeoxyglucose Positron Emission Tomography/Computed Tomography Angiography: Initial Results at an Infective Endocarditis Referral Center. *Circulation*.

[69] Arockiam AD, Agrawal A, El Dahdah J, Honnekeri B, Kafil TS, Halablab S (2023). Contemporary Review of Multi-Modality Cardiac Imaging Evaluation of Infective Endocarditis. *Life (Basel, Switzerland)*.

[70] Holcman K, Szot W, Rubiś P, Leśniak-Sobelga A, Hlawaty M, Wiśniowska-Śmiałek S (2019). 99mTc-HMPAO-labeled leukocyte SPECT/CT and transthoracic echocardiography diagnostic value in infective endocarditis. *The International Journal of Cardiovascular Imaging*.

[71] Juneau D, Golfam M, Hazra S, Erthal F, Zuckier LS, Bernick J (2018). Molecular Imaging for the diagnosis of infective endocarditis: A systematic literature review and meta-analysis. *International Journal of Cardiology*.

[72] Hyafil F, Rouzet F, Le Guludec D (2017). Nuclear imaging for patients with a suspicion of infective endocarditis: Be part of the team!. *Journal of Nuclear Cardiology: Official Publication of the American Society of Nuclear Cardiology*.

[73] Dursun M, Yılmaz S, Yılmaz E, Yılmaz R, Onur İ, Oflaz H (2015). The utility of cardiac MRI in diagnosis of infective endocarditis: preliminary results. *Diagnostic and Interventional Radiology (Ankara, Turkey)*.

[74] El Ouazzani J, Jandou I, Christophe Thuaire I (2020). Thrombus or vegetation?Importance of cardiac MRI as a diagnostic tool based on case report and literature review. *Annals of Medicine and Surgery (2012)*.

[75] Habib G, Badano L, Tribouilloy C, Vilacosta I, Zamorano JL, Galderisi M (2010). Recommendations for the practice of echocardiography in infective endocarditis. *European Journal of Echocardiography: the Journal of the Working Group on Echocardiography of the European Society of Cardiology*.

[76] Ho CB, Vejlstrup NG, De Backer O, Søndergaard L (2023). Intracardiac echocardiogram to diagnose infective endocarditis after transcatheter aortic valve-in-valve implantation. *Catheterization and Cardiovascular Interventions: Official Journal of the Society for Cardiac Angiography & Interventions*.

[77] Østergaard L, Vejlstrup N, Køber L, Fosbøl EL, Søndergaard L, Ihlemann N (2019). Diagnostic Potential of Intracardiac Echocardiography in Patients with Suspected Prosthetic Valve Endocarditis. *Journal of the American Society of Echocardiography: Official Publication of the American Society of Echocardiography*.

[78] Parra JA, Hernández L, Muñoz P, Blanco G, Rodríguez-Álvarez R, Vilar DR (2018). Detection of spleen, kidney and liver infarcts by abdominal computed tomography does not affect the outcome in patients with left-side infective endocarditis. *Medicine*.

[79] Vitali P, Savoldi F, Segati F, Melazzini L, Zanardo M, Fedeli MP (2022). MRI versus CT in the detection of brain lesions in patients with infective endocarditis before or after cardiac surgery. *Neuroradiology*.

[80] Mikail N, Benali K, Ou P, Slama J, Hyafil F, Le Guludec D (2015). Detection of Mycotic Aneurysms of Lower Limbs by Whole-Body (18)F-FDG-PET. *JACC. Cardiovascular Imaging*.

[81] Iung B, Doco-Lecompte T, Chocron S, Strady C, Delahaye F, Le Moing V (2016). Cardiac surgery during the acute phase of infective endocarditis: discrepancies between European Society of Cardiology guidelines and practices. *European Heart Journal*.

[82] Braghieri L, Kaur S, Black CK, Cremer PC, Unai S, Kapadia SR (2023). Endocarditis after Transcatheter Aortic Valve Replacement. *Journal of Clinical Medicine*.

